# Supplementary material for: Homogeneous Incorporation of Gallium into Layered Double Hydroxide Lattice for Potential Radiodiagnostics: Proof-of-Concept
Source: Nanomaterials (Basel). 2020 Dec 26;11(1):44. doi: 10.3390/nano11010044 (PMC7824364; doi:10.3390/nano11010044)
Supplement: Supplementary file 1 [file nanomaterials-11-00044-s001.pdf]

nanomaterials

Supporting Information

Homogeneous Incorporation of Gallium into Layered Double Hydroxide Lattice for Potential Radiodiagnostics: Proof-of-Concept

Do-Gak Jeung [1], Tae-Hyun Kim [2,*] and Jae-Min Oh [1,*]

1 Department of Energy and Materials Engineering, Dongguk University-Seoul, Seoul 04620, Korea; jdk941101@naver.com

2 Department of Environmental Engineering, Seoul National University of Science and Technology, Seoul 01811, Korea

* Correspondence: th.kim@seoultech.ac.kr (T.-H.K.); jaemin.oh@dongguk.edu (J.-M.O.)

1. Crystal structure analysis

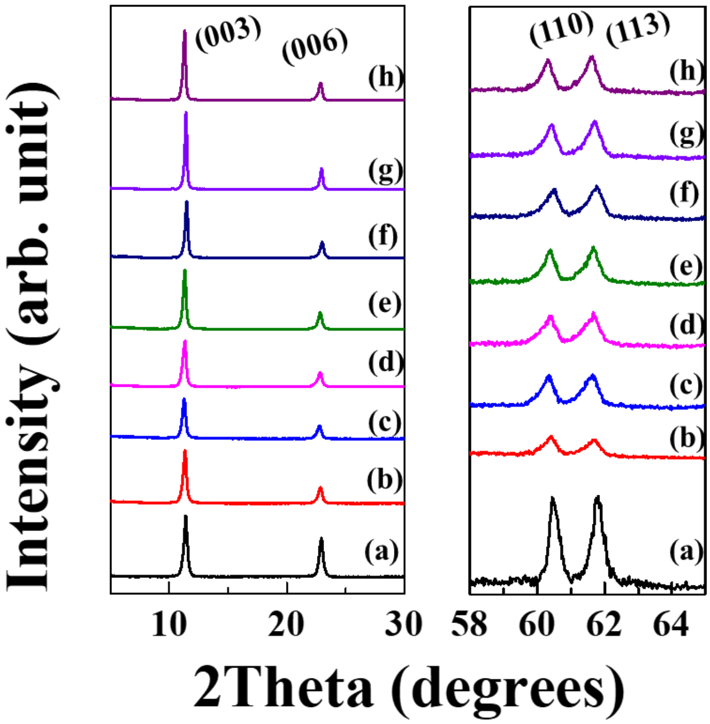

Figure S1. X-ray diffraction patterns of (**a**) parent MgAl LDH, (**b**) – (**h**) Ga@LDHs. (0.5, 1, 2, 3, 6, 12 and 24 h).

2. Morphology and surface properties

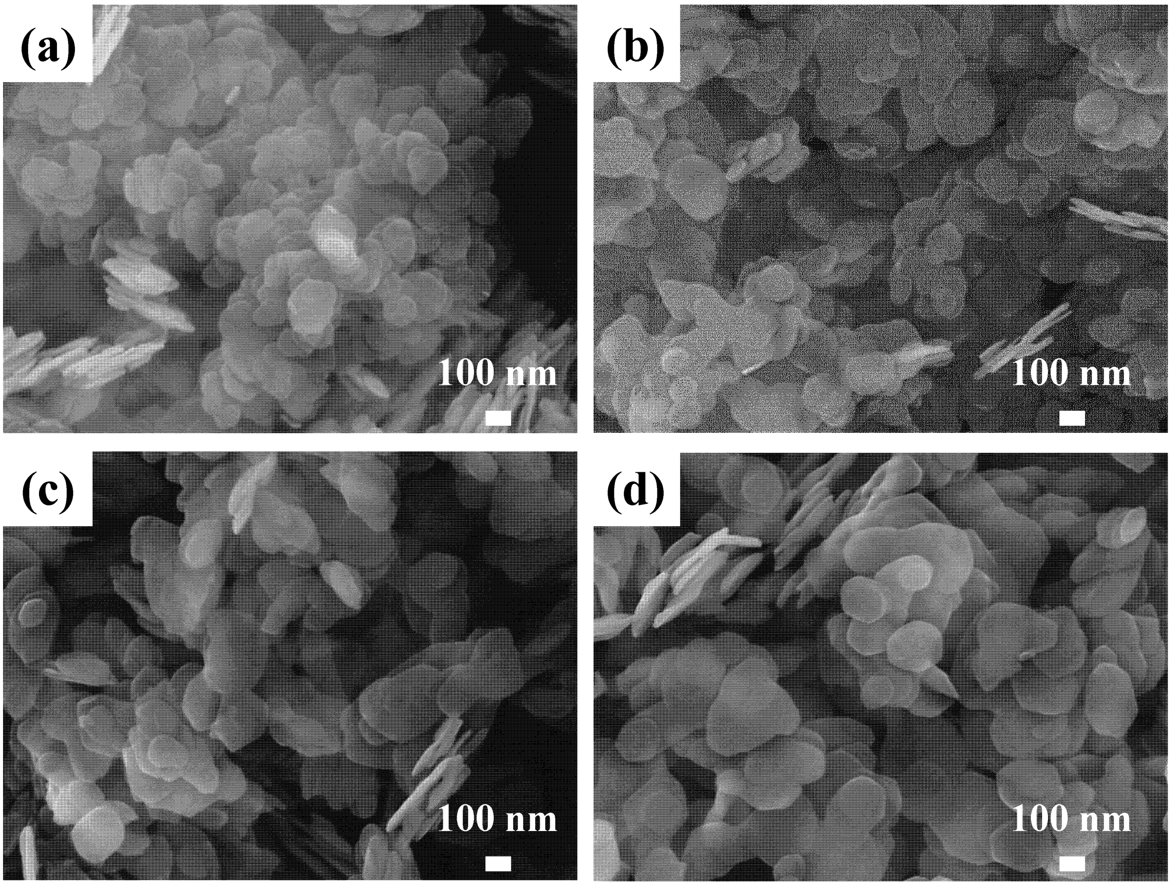

Figure S2. Scanning electron microscope image of (**a**) Ga@LDH-1, (**b**) Ga@LDH-3, (**c**) Ga@LDH-6 and (**d**) Ga@LDH-12.

3. Distribution of incorporated Ga^{3+} in Ga@LDH

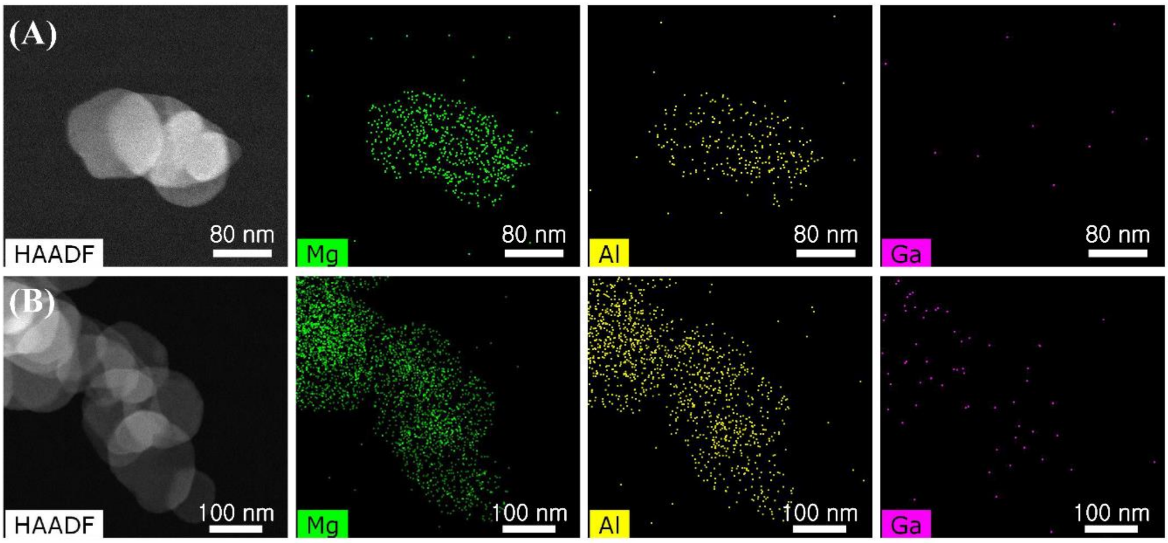

Figure S3. High-angle annular dark-field (HAADF) and element mapping images from scanning transmission electron microscope on (**A**) Ga@LDH-0.5 and (**B**) Ga@LDH-2.

4. Local environments of incorporated Ga^{3+} in Ga@LDH

For comparison, the MgGa-CO_3-LDH was synthesized by conventional co-precipitation. The mixed metal solution of $Mg(NO_3)_2 \cdot 6H_2O$ (0.315 M) and $Ga(NO_3)_3 \cdot xH_2O$ (0.105 M) was titrated with mixed alkaline solution of NaOH (1.2 M)/$NaHCO_3$ (0.126 M) until pH 9.5. The mixed solution was reacted with vigorous stirring at room temperature for 24 h. After then, the solid part was centrifuged, washed with deionized water and lyophilized.

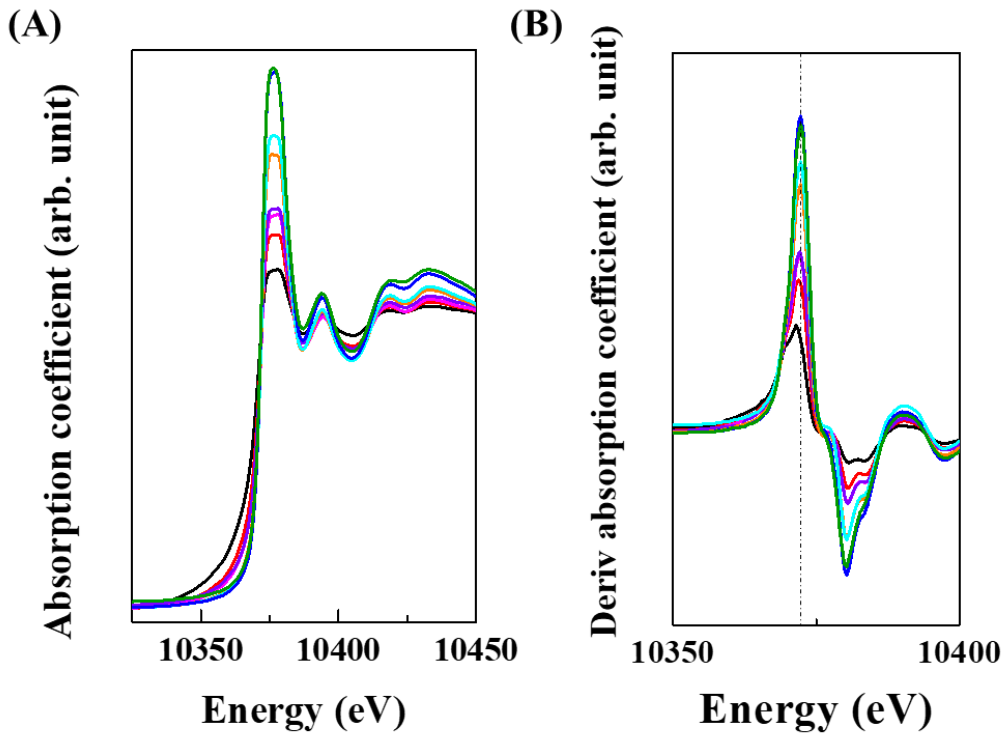

Figure S4. Ga K-edge X-ray absorption spectroscopy spectra of Ga@LDHs (black, red, magenta, violet, orange, cyan, blue line for 0.5, 1, 2, 3, 6, 12 and 24h) and MgGa-LDH (green line). (**A**) X-ray absorption near-edge structure and (**B**) derived X-ray absorption near-edge spectra.
